# Long-Acting Buprenorphine Formulations as a New Strategy for the Treatment of Opioid Use Disorder

**DOI:** 10.3390/jcm12175575

**Published:** 2023-08-26

**Authors:** Icro Maremmani, Maurice Dematteis, Edward J. Gorzelanczyk, Alessandro Mugelli, Stephan Walcher, Marta Torrens

**Affiliations:** 1 VP Dole Research Group, G. De Lisio Institute of Behavioural Sciences, Via di Pratale 3, 56121 Pisa, Italy; icro.maremmani@med.unipi.it; 2 UniCamillus, International Medical University in Rome, Via di Sant’Alessandro 8, 00131 Rome, Italy; 3 Department of Pharmacology and Addiction Medicine, Grenoble-Alpes University Hospital, Grenoble Alpes University, Rue de la Chantourne, 38043 Grenoble, France; maurice.dematteis@univ-grenoble-alpes.fr; 4 Department of Theoretical Basis of Biomedical Sciences and Medical Informatics, Nicolaus Copernicus University, Collegium Medicum, 85-067 Bydgoszcz, Poland; medsystem@medsystem.com.pl; 5 Faculty of Philosophy, Kazimierz Wielki University, 85-092 Bydgoszcz, Poland; 6 The Society for the Substitution Treatment of Addiction ”Medically Assisted Recovery”, 85-791 Bydgoszcz, Poland; 7 Department of Neurosciences, Psychology, Drug Research and Child Health (NeuroFarBa), University of Florence, Via della Pergola, 50121 Firenze, Italy; alessandro.mugelli@unifi.it; 8 CONCEPT Center for Addiction Medicine, Kaiserstrasse 1, D-80801 Munich, Germany; kontact@moviemed.de; 9 Addiction Research Group, Hospital del Mar Research Institute Barcelona, 08003 Barcelona, Spain

**Keywords:** long-acting buprenorphine medications, OUD particular settings, impact on OUD treatments

## Abstract

Long-acting buprenorphine formulations have been recently marketed for the Opioid Agonist Treatment (OAT) of opioid use disorder (OUD) associated with medical, social, and psychological support. Their duration of action ranges from one week up to 6 months. The non-medical use of opioids is increasing with a parallel rise in lethal overdoses. Methadone and buprenorphine are the standard treatment for opioid dependence. Methadone Maintenance Treatment (MMT) is widely recognized as one of the most effective ways of reducing the risks of overdose, crime, and transmission of HIV (Human Immunodeficiency Virus) in people who use opioids; however, its effectiveness has been hindered by low rates of uptake and retention in treatment. Furthermore, both methadone and buprenorphine are widely diverted and misused. Thus, a crucial aspect of treating OUD is facilitating patients’ access to treatment while minimizing substance-related harm and improving quality of life. The newly developed long-acting buprenorphine formulations represent a significant change in the paradigm of OUD treatment, allowing an approach individualized to patients’ needs. Strengths of this individualized approach are improved adherence (lack of peaks and troughs in blood concentrations) and a reduced stigma since the patient doesn’t need to attend their clinic daily or nearly daily, thus facilitating social and occupational integrations as the quality of life. However, less frequent attendance at the clinic should not affect the patient–physician relationship. Therefore, teleconsulting or digital therapeutic services should be developed in parallel. In addition, diversion and intravenous misuse of buprenorphine are unlikely due to the characteristics of these formulations. These features make this approach of interest for treating OUD in particular settings, such as subjects staying or when released from prison or those receiving long-term residential treatment for OUD in the therapeutic communities. The long-lasting formulations of buprenorphine can positively impact the OUD treatment and suggest future medical and logistic developments to maximize their personalized management and impact.

## 1. Current Standards in OUD Care

### 1.1. Principal Issues in OUD Patients

#### 1.1.1. Mortality

Opioid overdoses remain a foremost cause of death, especially among young Opioid Use Disorder (OUD) patients in Europe. Recent data show that it accounts for over 3.4% of all deaths among 15–40-year-old Europeans. Increased mortality is primarily related to overdose and is 15-fold higher in people injecting drugs [1,2]. Agonist Opioid Treatment (OAT), methadone or buprenorphine, effectively limits overdose mortality. However, evidence shows that mortality during and after OAT differs by type of drug and is significantly lower with buprenorphine than with methadone both in and out of OAT [3]. A high risk of relapse and mortality by overdose can occur after OAT short-term detoxication, as detoxified patients have significantly reduced or completely reset their opioid tolerance. A similarly elevated risk is present in patients who remained longer in the hospital for medical reasons outside of addiction and after prison and therapeutic community (TC) discharge [4].

#### 1.1.2. Health

In infectious diseases, 78% of HCV (Hepatitis C Virus) transmissions are attributable to subjects injecting drugs [5]; however, advances in HCV treatment have created a treatment opportunity for such individuals. Preliminary evidence shows that HCV treatment in OUD populations is effective, with results comparable with those of clinical trial populations. Despite the approval of new medicines for HCV, system/infrastructure/stigma hinders the access of OUD patients to HCV care.

Addiction care doctors and infectious disease/internal medicine specialist liver doctors should work together to address the HCV public health challenge. A correct application of a long-term OAT is necessary to treat HCV in opioid-addicted patients successfully. Opioid-addicted patients who inject or have injected opioids present the highest risk of reinfection; therefore, limiting the risk of infection in heroin-addicted patients whose HCV has been eradicated is vital. Heroin addiction is a chronic, relapsing disease and can occur in a severe form even after shorter or longer periods of abstention or after the end of active treatment [6]. Accordingly, we must treat OUD patients with ‘continuum care’ rules, as with all other chronic diseases [7]. In our opinion, the possibility of eradicating HCV leads to the need to rethink the methodology used in OATs. Limitations of time treatment, premature treatment interruption, and maintenance dosages lower than the blocking ones (harm reduction strategies) should be avoided in patients where the virus has been eradicated. If the patient relapses into intravenous drug addiction, there is a risk of reinfection [8].

#### 1.1.3. Unemployment

Problematic substance use increases the likelihood of unemployment [9]. According to the European Quality Audit of opioid treatment (EQUATOR) multicenter study, the proportion of individuals reporting full- or part-time employment varied across European countries [10]. Interestingly, more patients receiving buprenorphine-monotherapy or buprenorphine-naloxone were employed than those receiving methadone or slow-release oral morphine. Many OUD patients across Europe remain outside treatment, and not all those in treatment derive optimal benefits. The analysis showed that opioid-dependent people report high levels of polydrug use, high unemployment and past imprisonment rates, and significant physical and mental health comorbidities regardless of OAT. Treatment systems should be judged by their ability to reduce harm and promote individual recovery and social reintegration.

It is always difficult for addicts to find a job when it is also difficult for young, non-addicted people. What is clear is that work is less likely to be a practical option for patients requiring daily clinic visits or mobility difficulties.

#### 1.1.4. Crime

Eighty percent of people with OUD are involved in crime. Around half of the patients in and out of treatment have a history of imprisonment 3.4 times for drug-related offenses. This fact confirms that many patients and users have repeatedly encountered the criminal justice system due to the inter-relationship between drug dependence and crime [11].

Presently, OAT in prison provides continuity for patients receiving treatment before imprisonment and an excellent opportunity to recruit patients into the therapy [12]. However, despite OAT benefits in jail [13], opioid maintenance treatment is unavailable in several European countries. OUD inmate patients discontinuing OAT in prison are at higher risk of overdose, mortality and reoffending after leaving prison than patients continuing treatment [10,13].

### 1.2. OUD Treatment Is Available: Pharmacological and Psychosocial Interventions

The pharmacological treatment of opioid addiction aims to stop the action of heroin in the brain (heroin rewarding craving), stabilizing and maintaining opioid system activity. The main task of pharmacotherapy for heroin addiction is to ensure heroin use interruption and the resumption of a productive and satisfactory life [14,15,16,17]. Many drugs can be helpful to this aim, such as opioid agonists (methadone, LAAM, buprenorphine, slow-release morphine), opioid antagonists (naloxone, Naltrexone), a buprenorphine-naloxone combination, and finally, a depot naltrexone formulation. The pharmacological act of opioid agonists and antagonists may differ. Yet, there is a commonality from a clinical point of view: both substances can make the opioid receptors unresponsive to the heroin action by blocking them. Indeed, the dosage at which this phenomenon occurs is called the “blocking dose”, and patients taking the “blocking dose” know that an additional dose of heroin will no longer affect the brain. As a result, they will tend not to take it, endorsing de-conditioning from the reward (sense of pleasure) induced by heroin; eventually, this should lead to ending heroin use [15,18,19,20].

Heroin owes its addictive property to its “rewarding effect”, a feeling of pleasure after assuming a dose higher than an individual’s tolerance [21]: heroin addiction is characterized by maladaptive behaviors fulfilled by the patient to reach its reward and avoid discomfort due to the abstaining (dependence–withdrawal). In non-medical terms, addiction can be better defined as a state of obligation in which the subject is willing to pay a very high cost on the physical, behavioral and psychopathologic levels while continuing to use the substance [22].

In the pharmacotherapy of heroin-addicted persons, doctors should use, at distinct times, anti-withdrawal and blocking dosages of medications interfering with the opioid mechanism. One of the main problems of heroin addiction pharmacotherapy is that the anti-withdrawal dose, which prevents suffering after stopping use, usually does not correspond to the blocking dose (therapeutic dose). For the full agonist methadone, the blocking dose is considerably higher than the anti-withdrawal one; for the partial agonist buprenorphine, the difference between the two doses is lower, and for the antagonist naltrexone, the anti-withdrawal dose does not exist because this medication does not give relief for a withdrawal syndrome. Specifically, methadone expresses its receptor-blocking action gradually when increasing the dose. Upper 60 mg, the dose augmentation causes a progressive increase in the number of receptors blocked. Between 80 and 120 mg, it causes blockade of receptors in most patients, but higher doses may be necessary since methadone’s kinetics vary depending on the patient’s genetics. To reach the same receptor blockade, which depends on the methadone blood dose, oral doses can vary widely, achieving a 1:30 ratio [23,24,25]. Buprenorphine explicates blocking activity between 2 mg and 16 mg [26] by 36–50% and 79–95%, respectively [27], after which increasing doses do not significantly influence the number of blocked receptors but only intensify the drug’s duration; 50 mg of Naltrexone permanently block receptors, and higher doses are required exclusively for subjects who use a considerable amount of heroin. These differences are related to different receptor interaction modes. Methadone is a full agonist, and receptor stimulation is proportional to the dose. Buprenorphine is a partial agonist, and after a certain amount, the stimulation is not directly proportional to quantity (ceiling effect) [28,29,30]. Naltrexone does not stimulate opioid receptors and, in some ways, seems to be more of an inverse agonist than an antagonist [31]. Naltrexone can induce panic attacks even in people not tolerant to opioids [32].

Heroin-addicted patients must first stop heroin use and resolve their withdrawal syndrome by themselves or with medical assistance. The latter requires a prescription of withdrawal-suppressing doses of opioid agonists; then, patients should gradually enhance dosages of opioid agonists until the blocking quantity is attained to prevent the rewarding effect of sporadic heroin use. If a patient can stop heroin use without opioid agonist medications, then blocking doses of an opioid antagonist can be helpful. However, when using opioid antagonists, there is a risk that the lack of a feeling of well-being and the post-withdrawal sense of “discomfort” increases the risk of treatment breakdown. The accomplishment of opioid agonist therapy depends on the fact that it blocks heroin’s action but, at the same time, stabilizes subjects’ opioid systems that used to be heroin-stimulated and are unable to resume their function after heroin ends immediately. This lack of receptor stimulation is believed to be the leading cause of the poor efficacy of opioid antagonist treatments [33,34].

Individual/group and cognitive behavioral therapy are effective psychosocial treatment types combined with pharmacological treatments [35,36,37]. Integrated treatment programs involving pharmacological and psychosocial interventions are proven effective [38].

### 1.3. What Are the Current Challenges to Treatment?

#### 1.3.1. During the Engagement Process

Several critical challenges in treating opioid dependence are still open. First, engaging patients in treatment remains challenging; only 50% of people with OUD are involved in comprehensive therapies [39].

The fear of social stigma is one of the main reasons for low-level engagement in the treatment [40,41]. The worst obstacle to the effective treatment of heroin-addicted patients is the stigmatizing attitude, which may come from addiction practitioners and may be referred to as “iatrogenic stigma”, in which methadone or buprenorphine treatment is defined as “substitution treatment” or “replacement treatment”. In Dr. Dole’s experience, methadone was a behavior-normalizing drug that would rebalance the endogenous opioid system persistently damaged by harmful narcotics, such as heroin. For this reason, methadone must be administered at adequate dosages and as a maintenance regimen.

The treatment pathway is often hard to navigate, and many patients may be concerned about complying with OAT’s strict rules. OAT-providing physicians and pharmacists may be subjected to different sets of OAT regulations. German OAT is characterized by strict rules to ensure quality but with the questionable effect of making it difficult to dispense treatment and legally risky for the operators. The Belgian system is noteworthy since it pursues the integration of patients into standard medical practice and society itself. Connection with a sound support system, networking, regular education, and periodic evaluation of how the system works guarantee the best possible outcome for patients [42].

Patients may have failed previous therapy experiences. The ‘revolving door syndrome’ marks the concluding stage for patients with severe addiction; long-term OAT can be considered a valid reproduction of this condition. By contrast, treatment followed by early readmission (less than one month from the latest treatment) can be a proxy for the tendency to avoid implementing long-term treatment for heroin users. As suggested by the European Opiate Addiction Treatment Association (EUROPAD)-Radar system study, keeping the patient in treatment if possible, removing obstacles to chronic treatment, and the unification of treatment modalities across Europe remains the main challenge in drug addiction health policy for the near future [6].

Finally, many patients may not be ready to stop using drugs [43]. Harm Reduction politics are needed for these patients [44], and some particular OAT targets and features of dual disorder patients may be reasonable. Convergence on overlapping targets may be hypothesized if harm reduction and specific treatment share the same therapeutic instruments. Opioid agonists are also valuable as harm reduction instruments if harm reduction is conceived as treatment, but only at a low-threshold level. The personal and social impact of opioid agonist-mediated harm-reduction seems effective in higher-risk populations, such as dual disorder heroin-addicted patients, who have turned out to be sensitive to therapeutic opioid agonism. Harm reduction can best be regarded as a low-level approach to more severely disabled subjects, bridging the gap between the street and clinical settings by a sub-therapeutic but specific pharmacotherapy. Stepping up from harm reduction to a higher level of intervention should be the goal of harm reduction. Transitioning to a particular treatment is particularly important for dual disorder-addicted patients, who can be expected to receive a more significant benefit; without that transition, they are likely to quickly lose the opportunity to attain a positive outcome [45].

#### 1.3.2. During the Treatment

During treatment, patients can receive a suboptimal dose of medications [43,46]. In many countries, among AO-treated persons who inject drugs, medication dosages were suboptimal according to international guidelines. Poor adherence to international guidelines for opioid agonist therapies, aggressive law enforcement, and a lack of prison treatment must be addressed to optimize treatment and reduce harms associated with untreated OUD.

A recent review identified limited articles examining sub-optimal dosing in population groups. The results varied between papers but showed a high degree of low dosing. The study also looked at differences between the prescribing of methadone and buprenorphine to determine if the medications have a distinction between suboptimal rates. The authors concluded that suboptimal dosing for OAT found in the papers is commonplace in substance misuse services within the UK [47].

Often, patients cannot comply with the treatment regimen [43]. OAT is very unequally regulated in different countries. Opioids have a known non-medical use potential. Many rumors and reports are afloat on the outflow of substitutes to the black market and harmful up to fatal consequences of their uncontrolled use. It is comprehensible that societies try to avoid as much use as possible using regulations, also saving a high treatment quality. Most countries neither trust the doctors nor the patients and have put the treatment under strict rules and control. However, a few countries have gone nearly another way, disclaiming all these regulations. From the view of the regulating countries, treatment quality should be worse there; nevertheless, OAT-providing physicians report the opposite on all outcome parameters [42].

Patients’ misuse and diversion of OUD medications represent a severe public health problem and result in worsening outcomes with an increased risk to the individual’s health, a lack of progression in recovery and an increase in criminal activity [48]. Diversion of OAT has impacts on a community that is beyond the OAT recipient. The direct impact includes risk to others (unsupervised use; unintended exposure of children to diverted medication). The indirect implications consist of the economic costs of untreated opioid dependence, crime and loss of productivity. While treatment for opioid addiction is essential and must be supported, reducing misuse and diversion is vital to ensure the best possible care [49]. Three strategies to address misuse or diversion have been defined, depending on impact (effectiveness and ease of implementation)—recommended higher impact, other important strategies, and strategies not recommended relative to other options. Preferred methods include promoting access to treatment and using product formulations that are less likely to be misused. However, additional data and innovative approaches to address this complex problem are needed [50]

Patients may not be involved in the decision-making [43,46]. As patients and their informal caregivers have become increasingly involved and actively participating in the therapeutic process, though not at a decision-making level, in rehabilitation and prevention, it is crucial to provide information about the nature of the disease, its features and its course while clarifying which available treatments are the most effective and overcoming misleading thinking styles.

Nevertheless, addicted patients must be motivated to the treatment because patients’ thoughts, effects and behaviors are all displayed ambivalently. This observable ambivalence mirrors a psychopathological one, an expression of a “neurobiological” conflict in which addicted patients cannot counteract the symptoms of their disease. To neutralize this addictive ambivalence, a therapeutic alliance is needed. There is evidence that a solid therapeutic partnership predicts better outcomes in therapy since the patient feels comfortable with the therapist, has a sense of shared goals or purpose, and feels a sense of safety and belief in the therapy process [51].

Psychosocial support may be inadequate [52]. Treating opioid receptors with full or partial agonist medications for opioid-use disorder with psychosocial interventions is essential for patients who develop OUD. Still, no high-quality evidence currently exists to support any psychosocial treatment over standard care for remaining in treatment, reducing substance use or improving mental or global state, at least in people with serious mental illnesses and substance misuse [53]. The low incidence of psychopathological manifestations and the reasonable social adjustment of long-term methadone treatment subjects demonstrate its effectiveness on patients’ psychosocial adjustment. Still, psychosocial treatment has been recognized as a critical element of the patient’s positive outcome since the first publications of Dole and Nyswander [15,54].

Remarkably, the meaning of psychosocial features in drug addiction is often misunderstood as the core of the disease or independent indicators of global severity instead of as possible expressions and consequences of addictive psychopathology. Furthermore, evidence about the psychosocial impairment of drug-addicted patients is treated as if it were directly dependent on the theory and practice of psychosocially based treatment. Thus, we must avoid the paradox in which psychosocial requirements or engagement are employed as therapeutic instruments in treating a condition characterized by disrupting and neutralizing psychosocial resources [55].

Lastly, there is no continuity of care in prison environments. Although it can be sustained that the availability of treatment alternatives to imprisonment for drug dependence is a valuable policy option under various conditions and that this option is open to further improvement [56], continuing or initiating treatment in prison is not general politics worldwide [13].

Interventions against drug addiction aim to achieve an adequate level of individual well-being, which does not vary despite different starting situations. The prison system should implement medical skills that have proven effective in ensuring behavioral control and health preservation for not-imprisoned individuals. Agonist maintenance by methadone or buprenorphine is feasible within prison walls, using the same criteria adopted outside. Agonist drugs can allow a safer relationship with jailed individuals and improve the prospects for early release by conditioning adaptive behaviors. Different schedules are suitable for different grades of addictive severity, and less severe patients may be released as free individuals with an option of therapeutic parole. Extremely ill, addicted patients may benefit from the isolation of prison life as they are initiated and stabilized on therapeutic regimens during custody. Thus, the prison system can be crucial in leading addicted patients towards therapy [57].

## 2. Innovation in OUD Care—Options for the Future

What are the options for the future? OAT aims to minimize harm from illicit drug use while optimizing the quality of life for people with OUD. OUD treatment goals are to reduce/cease opioid use and prevent damage, reduce key symptoms, improve health, patient’s functional status, quality of life (QoL) and well-being, and reduce social consequences. We must enhance medication, behavioral therapy and recovery support services [58]. New long-term intervention strategies aimed at drug management and continuous therapy monitoring would complement the increase in therapeutic pressure that would undoubtedly lead to lasting patient benefits [59].

The possible solutions will have to intervene in patient engagement and during treatment; the barriers against an early and timely engagement in treatment are the low attractiveness of entering treatment, the high addiction-related stigma, the possibility that patients and doctors have different therapeutic goals, and especially the limited access to treatment worldwide prevent an early and timely engagement in treatment [43,60]. The future services should reduce the treatment burden, enhance staff consideration of patient input on decision-making and therapeutic goals and adopt a flexible treatment policy.

During treatment, the barriers to effective treatment are the use of a suboptimal dose, the misuse and diversion of opioid medications, the inconvenient treatment pathway, inadequate support and limited access to the treatment [43,46,52]. The solutions comprise tailoring therapy to patients’ long-term needs, adopting comprehensive case management, and using integrated, simple care with a reduced burden. In addition, the treatment plan needs greater flexibility with pressure to reduce the dose of opioid medications.

Can innovation improve treatment outcomes? Better data sharing with modern technologies may better integrate addiction facilities between prison and the community [61]. Technology-driven modalities are in progress to bring in affordable and on-demand health support. These technologies are already available to people with mental health conditions in the form of Tess or Woebot. Tess is a Mental Health Chatbot developed by clinical psychologists, offering self-help chats. In contrast, Woebot is an app that acts as an automated therapist when finding a real one is impossible because of logistical and financial issues. Moreover, new pharmacological options are represented by depot forms of OUD medications [62].

## 3. Depot Medication: Evidence of Efficacy

Innovative long-acting buprenorphine formulations are now available for treating opioid use disorder (OUD). The rationale is constantly releasing buprenorphine through various delivery systems, such as depot injections and subcutaneous implants [63,64]. The injectable depot is a pre-filled syringe administered subcutaneously by a healthcare professional, either weekly or monthly [65]. The injection can occur in different body areas (upper arm, gluteus, abdomen, or thighs) and provides an extended release of buprenorphine lasting for several days or weeks, according to the chosen formulation. The injectable depot was proven effective in a relevant placebo-controlled study, which found improved drug abstinence in patients receiving monthly buprenorphine depot injections and a safety profile consistent with other buprenorphine products [66]. For an even longer-lasting effect, patients may undergo a small subcutaneous implant inserted into the inner side of the arm to gradually release buprenorphine at a low plasma concentration over six months, after which the implant is removed [67]. The depot formulation and the subcutaneous implant have demonstrated comparable or greater efficacy in preventing illicit opioid use than sublingual buprenorphine [68,69].

Injectable depot and, in general, long-acting buprenorphine formulations drastically reduce the frequency of administration, promoting adherence to the treatment plan by avoiding the burden of daily dosing. These strategies improved patients’ quality of life and autonomy, allowing for more flexibility in their personal and professional routines [65,70,71]. Moreover, constant buprenorphine release will enable us to avoid the fluctuations typically associated with cyclic intake of sublingual therapy. Long-acting buprenorphine formulations may also address some other concerns related to self-administered formulations, such as diversion, unsupervised use, and accidental exposure in minors [65]. Regarding patient satisfaction, weekly and monthly subcutaneous depot showed improved outcomes compared to sublingual buprenorphine in a randomized clinical trial that used patient-reported outcomes to determine treatment satisfaction [72]. However, it is worth noting that patient knowledge and treatment acceptance are crucial in determining its therapeutic success [73].

Through long-acting formulations, clinicians can now access more treatment options for patients with OUD. This fact allows a more tailored approach and increased versatility for managing the treatment pathway (e.g., selecting monthly or weekly depot injection) [67].

## 4. Adverse Effects and Limitations of Long-Acting Buprenorphine Formulations

LA-BUP depots and implants share systemic adverse events (AEs) reported with oral (sublingual and lyophilizate) buprenorphine [48,66,67,68,69,74,75,76,77,78,79,80,81]. Although LA-BUP formulations are expected to have fewer AEs due to lower peak-to-trough fluctuations in blood levels and less hepatic first-pass metabolism (lower production of norbuprenorphine, an active metabolite that causes dose-dependent respiratory depression), they may provide particular AEs because of:

- The higher blood levels (average steady-state concentration) of buprenorphine with the highest dosages of LA-BUP depots compared to oral formulations and the long-lasting effects (depots and implant) due to the slight decrease in blood concentration [79,80,81,82,83,84]. Both can be problematic with some comorbidities and in terms of drug–drug interactions.
- The route of administration: subcutaneous depot and implant.
- The presence of excipients.

### 4.1. Adverse Effects

#### 4.1.1. Systemic Adverse Effects

With both LA-BUP depots at their highest dosages, buprenorphine blood levels can be higher than daily sublingual buprenorphine at 32 mg/day [80,82,84]. With such long-lasting high levels, LA-BUP should be used cautiously in patients with compromised respiratory function (e.g., chronic obstructive pulmonary disease), sleep apnea, liver disease and cardiac arrhythmia risk (QT prolongation). Moderate to severe hepatic impairment results in higher plasma levels, and buprenorphine may cause acute hepatitis. Buprenorphine is contraindicated in both severe respiratory and hepatic insufficiency [79,80,81,82,83,84]. Assessment of liver function before LA-BUP initiation and regular monitoring during treatment are recommended. Other clinical situations should be considered in practice: renal impairment, head injuries, increased intracranial pressure, hypotension, prostatic hypertrophy, urethral stenosis, adrenal and other hormonal disorders, etc.) [79,80,81,82,83,84]. As for oral buprenorphine, especially during treatment induction and dose adjustment, LA-BUP may induce sedation, impeding driving or operating machinery. Concomitant use of benzodiazepines or other CNS depressants (alcohol, gabapentinoids, etc.) increases the risk of sedation, respiratory depression, and death [79,80,81,82,83,84]. In case of overdose, take-home naloxone may be of value but higher than usual doses, and repeated administration may be necessary to compete with the high affinity of buprenorphine at the mu-receptors. The long-lasting effects of LA-BUP may require naloxone infusion and prolonged monitoring in a hospital setting [85]. With the slight decrease of high blood levels, other drug–drug interactions may be of concern: cytochrome P450 3A4 (CYP3A4) inhibitors (e.g., protease inhibitors leading to additional blood level increase although the impact seems limited in pharmacokinetic models) [86], serotonergic drugs (serotonin syndrome), QT-prolonging drugs, including alcohol, cocaine and amphetamine (cardiac arrhythmia), and other opioids. Buprenorphine may modestly increase the QT interval [87].

The risk of a significant QT increase appears more related to the associated factors than buprenorphine, even with LA-BUP at supratherapeutic doses [88]. Regarding interactions with other opioids, guidance has been proposed to initiate LA-BUP without precipitating opioid withdrawal and to manage pain requiring opioid analgesics (see pain management) [85,89,90]. Naltrexone and nalmefene, two opioid antagonists prescribed for alcohol use disorder, are contraindicated. Education of the patient and communication with the family members and healthcare professionals involved in the treatment are essential. For these various situations, depending on the assessed risk, it appears more cautious to use first or, if necessary, to transfer to oral formulation until buprenorphine impact has been evaluated, enabling more accessible dose adaptation and avoiding prolonged plasma levels if discontinuation is required [85].

#### 4.1.2. Adverse Effects at the Injection Site

In descending order, pain, pruritus, erythema, swelling, induration, and, less frequently, bruising and cellulitis may occur. For LA-BUP depot, care must be taken to avoid intravenous, intramuscular or intradermal injection. Upon contact with body fluids, intravenous buprenorphine forms a depot with risks of occlusion, tissue damage and life-threatening thromboembolic events [66,67,68,76,77,78].

For the implant, protrusion or expulsion of the implant, infection at the insertion or removal site, damage to nerves or blood vessels during insertion or removal procedure, implant migration, missing implant or partial implant may occur. However, serious complications (nerve damage, migration leading to embolism and death) are rare and may result from improper implant insertion in the upper arm [67,69,74,75,79,80].

#### 4.1.3. Toxicity Associated with Excipients

The various formulations of LA-BUP contain excipients that can lead to adverse events, such as hypersensitivity, that constitute a contraindication. N-methyl-2-pyrrolidone (NMP) is one of the excipients of the Buvidal^®^ Monthly and Sublocade^®^. The potential for NMP to induce embryofetal malformations has been shown in animals at higher exposures than those associated with LA-BUP [80,81,82]. Buvidal^®^ Weekly contains anhydrous alcohol, but levels are low (<0.1 g; a standard drink contains 8–14 g of pure alcohol according to the country) and not considered a concern for pregnant or breastfeeding women, for patients with liver disease or epilepsy [80,81].

In humans, besides considerations about excipients, there is a lack of data on the safety and effectiveness of LA-BUP formulations in pregnancy and breastfeeding. Therefore, LA-BUP should be used during pregnancy and breastfeeding only if the potential benefit outweighs the potential risk to the fetus and the baby [79,80,81,82,83,84,85].

### 4.2. Limitations

A limitation to the use of probuphine implant is undoubted to be found in the need for a short surgery both for the introduction and for the necessary removal of the implant; even if the surgical procedure is effortless and can be quickly learned, after a short training, even by general practitioners, internists and psychiatrists who are generally the medical specialties involved in the treatment of OUD patients. Another limitation to the use of probuphine is the blood concentration, which is not such that it can be used as a blocking dose in the stabilization phase of the treatment. Only patients already stable at a low drug dose, around 2–8 mg/daily, can be treated with probuphine. Finally, the fact that implants can be used only for two cycles of 6 months (12 months) may constitute a limitation in the setting of a chronic disease that usually requires several years of treatment to stabilize. However, this treatment can be a step in the therapeutic trajectory before transitioning patients to other galenic.

The sublocate formulation, injectable monthly, maintains stable blocking dosages after at least seven days of treatment with oral buprenorphine with mostly mild and non-treatment-limiting side effects, such as headache, constipation, nausea and itching at the injection site. Brixadi (US) or buvidal (EU/AUS) showed adverse reactions in less than 5% of patients, including injection-site pain, constipation, headache, nausea, injection-site erythema, pruritus, insomnia and urinary tract infections. In the treatment of heroin addiction, the establishment of a therapeutic alliance with the patient is vital for therapeutic purposes. Doctors and health professionals play an essential role in patient counseling that helps the patient follow the care and solve social adaptation problems during rehabilitation. The use of depot formulations should not adversely interfere with these therapeutic aspects. The risk is that the lower frequency of addiction medicine services by patients may negatively affect the therapeutic pressure needed during all phases of the treatment, preventing the recognition of slight deterioration in the patient’s clinical picture [91].

## 5. Pain Management

Pain is often overlooked in patients under opioid substitution, while 23 to 68% suffer from chronic pain [92]. Interestingly, with the increasing use of LA-BUP, the pain management question is frequently raised. Because of the pharmacological profile of buprenorphine, which competes with opioid analgesics (see the drug–drug interactions described above), healthcare professionals may feel uncomfortable treating pain in patients on LA-BUP, and patients may be afraid to be untreatable in case of severe pain. The strategy will depend on the clinical situation: acute vs chronic pain, pain mechanisms and intensity.

### 5.1. Acute Pain

In routine practice, in terms of symptomatic medications, paracetamol and/or non-steroidal anti-inflammatory drugs (NSAIDs) and/or topical treatment (e.g., lidocaine) can be sufficient for mild to moderate pain intensity. If not, it is essential to remember that buprenorphine and methadone have analgesic properties. Still, they require a divided daily dosage of 3–4 times to take advantage of their analgesic properties that last 4–8 h [93]. Therefore, as proposed in situations that require transient dose adjustment with supplemental oral buprenorphine on top of LA-BUP (treatment initiation, delayed/missed dosing, psychological deterioration, etc.) [79,85], additional oral buprenorphine given 3–4 times per day could be an option to manage acute pain in patients treated with LA-BUP (off-label use). For more severe nociceptive pain (e.g., trauma, bone fracture, intensity ≥7 on a 0–10 rating scale) that requires strong opioid analgesics, it is only a matter of pharmacological competition [85,94]. Oral or intravenous short-acting opioid analgesics (e.g., immediate-release morphine, oxycodone, fentanyl) should be cautiously titrated up to the desired analgesic effect, with higher than usual dosages since patients are opioid tolerant. Patients should be monitored under the supervision of a physician, with attention to consciousness and respiratory function, because overdose may occur when attempting to overcome buprenorphine partial agonist effects or when buprenorphine plasma levels are declining [79,81,82,85]. It is essential to keep in mind that even with 32 mg/day of sublingual buprenorphine, there are still mu-receptors available for other opioid agonists (not all receptors are occupied by buprenorphine) [95,96] and that pain may involve other opioid receptors and non-opioid systems. Based on a proper evaluation of pain mechanism, co-analgesics (corticoids, antispasmodics, anti-neuropathic drugs, etc.) and non-pharmacological approaches may be more suitable than opioids. If necessary and according to the situation’s complexity, multidisciplinary management by pain and addiction specialists can be helpful [97]. Patients should be advised to inform their relatives and healthcare professionals of their treatment with LA-BUP to obtain the most appropriate treatment, for example, in an emergency. For anesthesia, similar reasoning can be applied. For instance, sufentanil has a higher affinity than buprenorphine for mu-receptors [94].

### 5.2. Chronic Pain

Chronic pain (>3 months duration) is highly prevalent in the general population (>20%) and is even higher in patients under opioid substitution (23–68%) [92,98,99]. It is a very heterogeneous situation that requires identifying the underlying mechanisms to propose the most appropriate treatment [100,101]. Numerous physicians follow the WHO analgesic ladder to choose analgesic drugs according to pain intensity. However, this famous ladder, developed to provide adequate pain relief for cancer pain (often a combination of nociceptive and neuropathic pain), is not adapted for chronic non-cancer pain [102,103]. Instead of intensity, assessment of pain mechanism is more relevant to providing the best medication [104]. For neuropathic pain (e.g., peripheral neuropathy), first-line medications are antidepressants (serotonin-noradrenalin reuptake inhibitors (SNRIs), tricyclics) or antiepileptics (gabapentinoids) [104,105,106]. Because tramadol is a mixed drug with both aminergic and opioidergic activities, it is proposed as a second-line medication, while potent opioids are offered only as third-line medications [105,106]. For nociplastic pain (e.g., fibromyalgia, low back pain), first-line medications are antidepressants (SNRIs or tricyclics), while opioids should be avoided [104,107]. In patients treated by LA-BUP, the same approach should be proposed [85]. Alternatively, transfer to methadone could be an option to treat patients with resistant complex chronic pain syndrome. Its pharmacological profile combines opioid, ketamine-like (NMDA receptor antagonist) and aminergic activities, which can be effective for different types of pain [108,109]. In any case, treatment of pain etiology and comorbidities, particularly psychiatric comorbidities and sleep disorders (insomnia, sleep apnea) that affect more than half of chronic pain patients, are essential [110,111,112]. Besides medications, non-pharmacological approaches are just as critical [101,104,105,113]: life hygiene (sleep, exercise, food hygiene, weight loss), relaxation, mindfulness, psychotherapy, such as cognitive behavioral therapy, physiotherapy, ergotherapy, transcutaneous electrical nerve stimulation (TENS), etc. In this context, clinical stability provided by LA-BUP could be helpful. Like for treating addictive disorders, this multidimensional and multimodal approach aims at improving the functional autonomy of patients, allowing them to achieve personal life goals and projects for a better quality of life, even if the pain is not entirely suppressed [114,115].

## 6. Expert Opinion: Making Decisions about OUD Care in the Future

### 6.1. How Do the Limits of Current Therapies for Treating OUD Interfere with the Healing Path and Patients’ Lives?

Patients’ access to treatment is crucial for treating OUD, regardless of their country of origin. Therefore, the new strategies must first facilitate access to care. In some cases, the availability of specialized centers and the number of professionals licensed to prescribe drugs is reduced, and patients may find OAT restrictive rules challenging. Clinicians must be flexible, proposing new approaches to meet patients’ needs. The first goal must be to start therapy gradually and progressively so patients experience greater well-being and stability, making even more restrictive rules acceptable in continuing the treatment path.

Considering the harm reduction strategy, initiating therapy is preferable to help the patient access treatment. The time for patients to improve is subjective. A flexible method must be developed to combine different approaches and allow patients to understand and accept the program in progress. Opening a perspective of change and improvement will enable individuals to project themselves differently in the future.

Finally, low-threshold treatments can be implemented using new therapeutic formulations; our clinical realities will provide further information and stimuli for using them.

Since each patient is unique, adopting a precision medicine approach to suit one’s needs is crucial to the therapy. Until recently, we have had minimal treatment options in our drug toolkit. Buprenorphine has been introduced in addition to methadone, but the therapeutic approaches are very similar. Using depot formulations or implants pushes us to reconceptualize treatment: the same active ingredient (buprenorphine) is administered differently, through the subcutaneous implant or by injection. As mentioned, engaging patients in treatment is one of the limitations of replacement therapies in use. The new pharmacological approaches must represent a resource that favors access to care. Therefore, from the addiction specialist’s point of view, identifying practical tools to implement the new treatment strategy in daily clinical practice is essential; from patients’ and their families’ perspectives, awareness of the advantages of this new delivery system is also vital. Proposing a more suitable treatment for a portion of previously unreachable patients would determine a more significant number of treatments delivered, benefiting a wider population. Secondly, we need to investigate the technologies to initiate such therapies and the possibility of offering them in the early stages of treatment, not only to stabilize patients.

Restabilized treatment is another crucial aspect that could be improved with new strategies. Although the medications are very effective, many patients drop out of treatment. Future observation may provide more information on the new formulations’ impact on the current treatments’ limits. Buprenorphine implant is not a new medication but a new way to administer the drug that could contribute to retaining patients in treatment.

### 6.2. What Are the Benefits of Treatment with the Buprenorphine Depot/Implant for Patients and the Care System?

Buprenorphine has widely demonstrated its efficacy and effectiveness for the OAT of OUD. We can advance the treatment of our patients with the pharmacokinetic properties of the buprenorphine depot/implant.

The depot/implant buprenorphine could have several advantages. The first is represented by access to a new treatment, both for patients who are not yet taking OAT and for patients who are in treatment and want to free themselves from the use of heroin definitively. The second advantage is that, with slow-release formulations, we can provide proper treatment with adequate dosages from the first administration and for prolonged times—a key point for OAT effectiveness. Since patients can reduce or increase the prescribed amount, the long-acting formulation can facilitate adherence to treatment with adequate dosages for a sufficient time, which is critical in OUD remission and relapse prevention. The third advantage is related to the fact that the long-acting formulation also reduces the frequency of access to the service for the single one dispensing the medication, leaving greater freedom to patients who can go to therapy and multidisciplinary psychotherapy services, reducing stigma and easing patients’ therapeutic adherence. Telemedicine may help maintain constant contact with the patient, evaluating the general conditions and emotional states.

In the United Kingdom, doctors have a financial incentive to reduce drug dosages, clinical supervision and duration of treatment to save from the economic management of Addiction Services. This situation is hazardous since encouraging doctors to prescribe only half or half doses of antibiotics or antihypertensive drugs would be a mistake. An advantage of implant or depot formulations is the expansion of therapeutic choices.

Some of the properties of the new formulations and some areas of clinical use can only be verified over time with direct experience. The treatment methods will change, and there will be no more need for the patient to attend the service, often the delivery of the drug, as it has been to date. Our leads are to re-negotiate the contact methods with the doctor and service. If patients’ freedom increases, we should also consider the opportunities for involvement in exploring other issues and the possibilities for change. This aspect must be clearly explained in the relationship with patients to facilitate their adherence to the therapy, which is multidisciplinary. In addition, the organization of Services should adapt to new needs, and doctors will have to develop new skills concerning the modalities of implant insertion.

Half of the patients do not receive adequate treatment for chronic pathologies of any nature. With depot/implant buprenorphine, we can help our patients achieve excellent stability in taking the correct dosage to achieve lasting clinical stabilization, a protective factor against possible relapses. However, patients often reject high dosages, which cause a perception of themselves as “more severe”, even more so if needed for prolonged periods. Another benefit of long-lasting clinical remission is the slightest request for psychosocial support, thanks to achieving good functioning through depot/implant buprenorphine therapy. These patients will be able to be followed by the general practitioner. If necessary, they will go to the specialist, reducing the workload in addiction clinics and enabling better resource management. Introducing this new modality leads us to reconsider the treatment modalities of each patient based on individual characteristics. Moreover, buprenorphine long-acting formulations could also be of interest to simplify the detoxification process, which is always very difficult and often not possible. Finally, this route of administration detaches the patient from the usual daily intake and removes the dependence on the everyday ritual gesture, providing a real advantage on many occasions.

### 6.3. From the Point of View of the Organization of Services for Treating Addictions in Different Countries: Can the Benefits Overcome Any Barriers to the System?

Addiction services in Spain are organized in a network parallel to that of mental health and general health services. In Spain, the buprenorphine implant must be inserted by a surgeon on an outpatient basis. Therefore, the organization of the services will have to change. The model already used could be again proposed for treating HCV, with weekly visits by the hepatologist at the addiction clinic, where all patients are collected from the area suffering from HCV. This type of organization works very well, both from the patient and organizational points of view. We can propose this model again if the surgeons give us their availability. Otherwise, a certain number could be collected of people to accompany the surgery for implant insertion. It is essential to ensure the accompaniment of patients by addiction service workers, representing trusted people with whom the patient has a meaningful therapeutic relationship. In addition, collaboration with anaesthesiologists’ pain therapy centers could be a resource for implant placement and removal operations. These organizational strategies can increase therapy costs for the Ministry of Health. Compared to the social costs determined by the reduction of crime among stabilized patients, savings do not directly affect the Ministry of Health but that of Justice, so it could be considered a slight advantage from the point of view of the organization of services. Organizational aspects and the cost increase must be well evaluated.

As for the organizational aspects, simplifying the perspective of implant placement is helpful. As for the implant of estrogen-progestins for contraceptive purposes, buprenorphine implantation will not represent a complicated operation. We must propose an easy way to use the treatment to eliminate the stigma related to the type of drug and facilitate compliance. The implant and depot formulations guarantee the correct therapy intake, clinical stabilization, and the possibility for general practitioners to follow up with patients more efficiently. This fact implies revising the organization of services with greater flexibility and availability of specialists to deal with the most severe and complex cases. These new formulations open perspectives, and the benefits overcome organizational barriers.

Introducing the buprenorphine long-acting formulations as a therapeutic tool for opioid addiction offers a challenge, uncertainty, and hope. The challenge concerns the treatment costs and organization: collaboration with other specialist services will be crucial for using implants. As past examples of multidisciplinary collaboration, the employment of anesthetists for electro-convulsant therapy in Psychiatry in Italy or the partnership between addiction services, HCV therapy specialists, and pain therapy in Spain is worth mentioning. The analogy with the use of the contraceptive implant is probably closer to the situation of our addiction services. All these organizational proposals will be the subject of discussion and will change the type of services we provide.

## 7. Conclusions

In conclusion, the introduction of depot/implant buprenorphine will improve the treatment system of OUD patients. Specifically, it will allow the following:

- Easier to implement precision medicine and personalization of opioid addiction treatments.
- Facilitating access to care and engagement of patients in treatment.
- Correct the assumption of the prescribed dosages for adequate time to achieve stabilization with symptoms remission and relapse prevention.
- Better patient retention in treatment.
- Greater freedom for patients, who are not forced to access the service frequently for taking medications.
- Greater freedom for therapists, who can better distribute resources.
- Reduction of the stigma associated with substance addiction and services deputies to care.
- Save social costs thanks to reducing the complications of untreated pathology (increased crime and comorbidity).
- Re-conceptualization of the Treatment and acquisition of new expertise by addiction specialists.
- Future possibilities of expanded use of depot/implant buprenorphine, as well as low-threshold therapies and harm reduction.

## Data Availability

Not applicable.
